# Is a Single Nephrographic Phase Computed Tomography Sufficient for Detecting Urothelial Carcinoma in Patients with Visible Haematuria? A Prospective Paired Noninferiority Comparison

**DOI:** 10.1016/j.euros.2023.06.005

**Published:** 2023-07-19

**Authors:** Kristina F. Galtung, Peter M. Lauritzen, Gunnar Sandbæk, Dag Bay, Erica Ponzi, Eduard Baco, Nigel C. Cowan, Anca M. Naas, Erik Rud

**Affiliations:** a Department of Radiology, Oslo University Hospital, Oslo, Norway; b Department of Life Sciences and Health, Faculty of Health Science, Oslo Metropolitan University, Oslo, Norway; c Institute of Clinical Medicine, Faculty of Medicine, University of Oslo, Oslo, Norway; d Department of Research Support for Clinical Trials, Clinical Trial Unit, Oslo University Hospital, Oslo, Norway; e Oslo Center for Biostatistics and Epidemiology (OCBE), Department of Biostatistics, University of Oslo, Oslo, Norway; f Department of Urology, Oslo University Hospital, Oslo, Norway; g Department of Radiology, Portsmouth Hospitals University NHS Trust, Portsmouth, UK; h Department of Pathology, Oslo University Hospital, Oslo, Norway

**Keywords:** Haematuria, Multidetector computed tomography, Prospective study, Transitional cell carcinoma

## Abstract

**Background:**

There is uncertainty about the utility of multiphase computed tomography (CT) compared with single-phase CT in the routine examination of patients with visible haematuria (VH).

**Objective:**

To compare the accuracies of single nephrographic phase (NP) CT and four-phase CT in detecting urothelial carcinoma (UC).

**Design, setting, and participants:**

This was a single-centre, prospective, paired, noninferiority study of patients with painless VH referred for CT before cystoscopy between September 2019 and June 2021. Patients were followed up for 1 yr to ascertain UC diagnosis.

**Intervention:**

All patients underwent four-phase CT (control), from which single NP CT (experimental) was extracted. Both were independently assessed for UC.

**Outcome measurements and statistical analysis:**

The primary outcome was the difference in accuracy between the control and experimental CT using a 7.5% noninferiority limit. Histologically verified UC defined a positive reference standard. Secondary outcomes included differences in sensitivity, specificity, negative (NPV) and positive (PPV) predictive values, and area under the curve (AUC). All results are reported per patient.

**Results and limitations:**

Of the 308 patients included, UC was diagnosed in 45 (14.6%). The difference in accuracy between the control and experimental CT was 1.9% (95% confidence interval −2.8 to 6.7), demonstrating noninferiority. Sensitivity was 93.3% versus 91.1%, specificity was 83.7% versus 81.8%, NPV was 98.7% versus 98.2%, PPV was 49.4% versus 46.1%, and AUC was 0.96 versus 0.94 for the control versus experimental CT. Limitations included a low number of UC cases and no definite criteria for selecting a noninferiority limit.

**Conclusions:**

The accuracy of NP CT is not inferior to that of four-phase CT for detecting UC.

**Patient summary:**

This study shows that a computed tomography (CT) examination with only one contrast phase is no worse than a more complex CT examination for detecting cancer in the urinary tract among patients presenting with visible blood in the urine.

## Take Home Message

Single nephrographic phase computed tomography (CT) is no worse than four-phase CT for detecting urothelial carcinoma among patients presenting with visible haematuria. Implementing a simplified CT protocol in the evaluation of these patients will not only decrease radiation exposure for patients, but also enhance the efficiency of radiological services.

## 1 Introduction

Visible haematuria (VH) is a frequent symptom requiring an extensive clinical workup, including cystoscopy for assessing the bladder, and urine cytology in combination with computed tomography (CT) for assessing the upper urinary tract [1]. According to a recent systematic review, patients presenting with VH have bladder urothelial carcinoma (UC) in 14–20%, upper tract UC (UTUC) in 0.4–1.2%, and renal cell carcinoma (RCC) in 1–2% [2]. Although the primary purpose of CT in the workup of VH is to detect UTUC, studies have shown promising results for detecting bladder UC too [3], [4].

A CT examination of the urinary tract may include different combinations of the unenhanced (UE), corticomedullary phase (CMP), nephrographic phase (NP), and excretory phase (EP) acquisitions, resulting in many different CT protocols.

Studies applying different CT protocols have shown 94–100% accuracy for detecting UTUC and 82–97% for detecting bladder UC [3], [5], [6], [7], [8], [9]. One study reported that the CMP was superior for detecting bladder UC, while others have shown that the NP was sufficient for assessing UTUC [6], [7], [10]. On the contrary, the French Society of Genitourinary Imaging favours a split bolus in the workup of VH [11]. The variation in patient selection and CT protocols in previous studies results in limited generalisability and difficulties when comparing results, and there is currently no consensus regarding the optimal CT protocol for detecting UC.

In a retrospective study, we showed that the NP was the only phase detecting all UTUCs, which necessitates confirmation in a prospective study [7]. Thus, this study aimed to prospectively compare the accuracy of single NP CT with that of four-phase CT for detecting UC in patients presenting with VH.

## 2 Patients and methods

According to the Norwegian Directorate of Health, VH qualifies for a standardised workup with CT, cystoscopy, and urine cytology for detecting UC unless there is a clinical suspicion of stone disease or cystitis. CT was defined as the first-line examination in this workup between September 2019 and June 2021. In this period, all patients with VH referred for CT before cystoscopy were assessed for eligibility.

### 2.1 Inclusion and exclusion criteria

The inclusion criteria were age >18 yr and at least one occasion of painless VH. The exclusion criteria were a cystoscopy within 6 mo prior to CT, previous or known UC, symptomatic urinary tract infection relieved by antibiotics, symptomatic stone disease, recent catheterisation or instrumentation, estimated glomerular filtration rate <30 ml/min/1.73 m^2^, allergy to iodinated contrast media, unable to provide consent for any reason, or no wish to participate for any reason.

### 2.2 Study design and ethical approval

The PROspective Trial for Examining Hematuria using Computed Tomography (PROTEHCT) was a single-centre, prospective, noninferiority, paired comparison of two CT protocols for assessing UC, RCC, and urinary stone disease in patients with VH. All patients signed a letter of consent, and the study was approved by the regional ethics committee (2019/395) and registered at ClinicalTrials.gov (NCT04077359). In this first paper, we report the results for UC. The results for RCC and stone disease will be reported in separate papers.

### 2.3 Outcomes

The primary outcome was the difference in overall accuracy between single NP (experimental) and four-phase (control) CT for detecting UC.

The secondary outcomes were the differences in sensitivity, specificity, false negative rate, false positive rate, negative predictive value (NPV), positive predictive value (PPV), and area under the curve (AUC).

### 2.4 Sample size estimation

We based the sample size calculation on restricted maximum likelihood statistics derived from the study of Liu et al. [12], a method used to assess equivalence and noninferiority in paired diagnostic tests. We used a one-sided 7.5% noninferiority limit to assess the difference in the accuracy of paired binary outcomes compared with a reference standard. The difference in accuracy was defined as the proportion of discordant pairs, that is, the difference in the proportion of patients receiving the correct diagnosis by either the control CT only or the experimental CT only. With the assumption of 5–10% discordant pairs, a power (*β*) of 80%, and a one-sided significance level (*α*) of 0.025, the trial required a minimum of 200–229 patients to exclude a difference in favour of the control of >7.5%. To allow for loss to follow-ups and dropouts, we aimed to include 300 patients. A team of medical statisticians at the Clinical Trial Unit reviewed the protocol and outcomes and performed sample-size estimation.

### 2.5 CT protocol

**Fig. 1:**
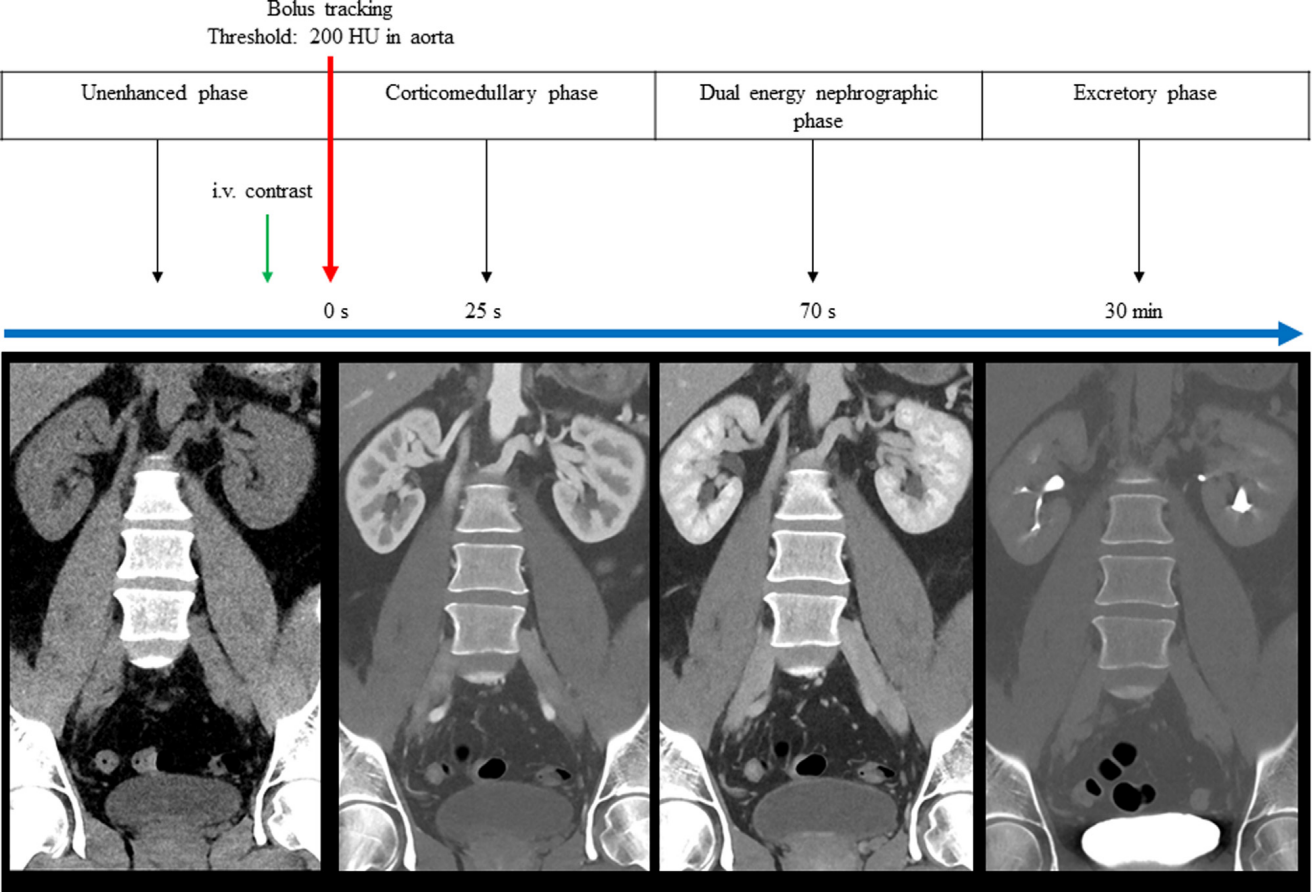
The CT protocol. All patients were asked to drink 1 l of water and not to void urine within 1 h before the CT. The contrast phases were acquired using bolus tracking with a 200 HU threshold in the abdominal aorta. The corticomedullary phase was performed at 25 s and the nephrographic phase at 70 s after the threshold. After the nephrographic phase was completed, patients got off the CT table and were asked to void urine. The excretory phase was performed at 30 min after the threshold. CT = computed tomography; HU = Hounsfield units; i.v. = intravenous.

All patients underwent four-phase CT (UE, CMP, dual-energy NP, and EP). Iohexol 350 mg/ml (Omnipaque GE Healthcare AS, Oslo, Norway), 2 ml/kg body weight, was administrated intravenously at 4 ml/s. All scans were acquired in the supine position from the upper kidney pole to the pelvic floor (NP from the diaphragm to the pelvic floor) using a 128-multidetector Somatom Definition Edge CT scanner (Siemens Healthineers, Forchheim, Germany) and reconstructed in the axial, sagittal, and coronal planes (Fig. 1).

### 2.6 CT reporting

For each patient, the four-phase CT was separated into control (all four phases) and experimental (NP only) CT. Three radiologists with 5–13 yr of experience in uroradiology formed a primary reading team, and two radiologists with >20 yr of general radiology experience formed a secondary team. By flipping a coin, two readers from each team were randomly assigned to independently score either the control or the experimental CT. The suspicion of UC in the bladder, ureter, and renal pelvis was prospectively scored using a five-point Likert scale. A Likert score of ≥3 defined positive CT. For the control CT, each phase was scored individually in the following order: CMP, NP, and EP. The highest score in any of the contrast-enhanced phases defined the overall Likert score. The UE was not scored for UC. All readers were blinded to the other readers’ CT reports and the cystoscopy and ureterorenoscopy (URS) reports.

### 2.7 Cystoscopy, urine cytology, and ureterorenoscopy

#### 2.7.1 Cystoscopy

A staff resident or a consultant urologist performed a flexible cystoscopy after CT. A transurethral resection of the bladder (TURB) was performed in case of a visible tumour. The primary team’s control CT report was available to the urologist at cystoscopy.

#### 2.7.2 Urine cytology

In the case of a negative cystoscopy, urine was collected and scored according to the Paris classification (I–VII) [13].

#### 2.7.3 Ureterorenoscopy

URS was performed when the primary control CT suspected UTUC, and/or visible blood from the ureter at cystoscopy and/or the Paris IV–VI urine cytology result. A biopsy was obtained when relevant.

### 2.8 Clinical follow-up

The follow-up time was 1 yr after the initial workup. If VH persisted, patients returned for repeated workups.

### 2.9 Reference standards

UC confirmed a histologically defined positive reference standard. In addition, in patients unfit for biopsy and/or surgery, the reference standard was positive if the control CT was positive, and the patients were clinically treated as having UC after multidisciplinary consensus. The reference standard was negative if no UC was diagnosed at the initial workup and during the follow-up.

### 2.10 Statistical analysis

Liu et al.’s [12] statistics were used to calculate the 95% confidence interval (CI) of the difference in accuracy between the control and experimental CT. Noninferiority was concluded if the 95% CI of the difference in overall accuracy for the primary reading team did not exceed 7.5% in favour of the control CT. We used descriptive statistics to calculate the sensitivity, specificity, accuracy, and predictive values with 95% CIs according to Wilson [[29]. The Newcombe [14] 95% CIs of the differences in sensitivity, specificity, and predictive values were reported. The “number of patients needed to scan” with the control CT to find one additional UC compared with the experimental CT was calculated as $\frac{1}{\Delta sensitivity\times prevalence}$. The AUC was calculated using the reference standard as the state variable and the Likert scores in the bladder, ureter, and renal pelvis as test variables. The AUCs were visualised in receiver operator characteristic curves, and any differences in AUCs were analysed according to the method of DeLong et al. [15]. The median age in those with and without UC was compared using the Mann-Whitney U test, and a *p* value of <0.05 was considered significant. We used IBM SPSS Statistics for Windows, version 28.0 (IBM, Armonk, NY, USA); MedCalc for Windows, version 20.009 (MedCalc Software, Ostend, Belgium); and R version 3.6.1 for statistical analyses.

## 3 Results

**Fig. 2:**
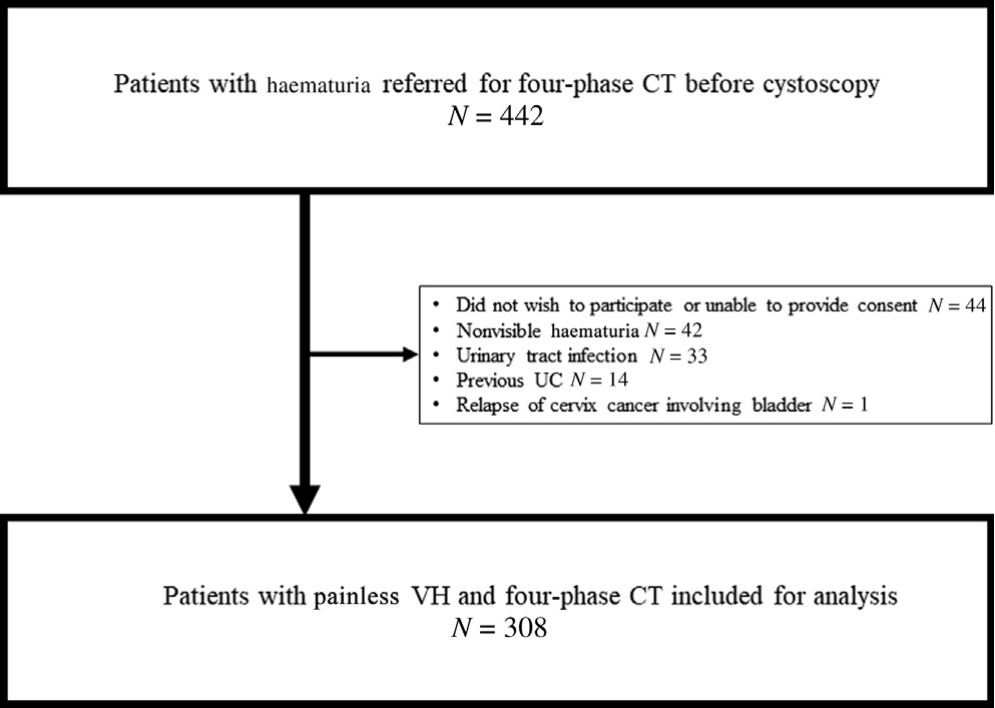
Flow diagram for the study. CT = computed tomography; *N* = number of patients; UC = urothelial carcinoma; VH = visible haematuria.

From September 2019 to June 2021, 308 patients met the inclusion criteria and were included in analyses (Fig. 2). The median age was 68 yr (interquartile range [IQR] 53–77, range 18–96), and 81% (250) were male.

After CT, 280 (91%) underwent cystoscopy, of whom eight (2.9%) underwent additional URS. At the initial workup, 14.6% (95% CI: 11–19) had UC, of whom 93.3% were male. The incidences of bladder UC and UTUC were 13.0% (95% CI: 10–17) and 1.6% (95% CI: 1–4), respectively. No patients had synchronous UTUC and bladder UC. The median ages of those with and without UC were 76 yr (IQR 67–82) and 67 yr (IQR 50–76), respectively (*p* < 0.001). After the initial workup, no UCs were detected during a 1-yr follow-up. The tumour characteristics of all UCs are summarised in Table 1.

**Table 1:** Tumour characteristics of all UCs, and the UCs missed on CT

All UCs			UC missed on CT			
			Primary reading team		Secondary reading team	
			Control CT	Experimental CT	Control CT	Experimental CT
	*N*	%	*n*	*n*	*n*	*n*
Bladder UC						
pTis	1	2.5				1
pTa	25	62.5	1	2	4	4
pT1	7	17.5	1		2	1
pT2	5	12.5				
Unknown pT	2	5.0		1	1	1
Total	40	100	2	3	7	7
UTUC						
pTa	1	20.0				
pT1	1	20.0	1	1	1	1
pT2	1	20.0				
pT3	1	20.0				
Unknown pT	1	20.0				
Total	5	100	1	1	1	1

CT = computed tomography; *N* = number of patients with UC; *n* = number of patients with missed tumour; pT = pathological tumour stage; UC = urothelial carcinoma; UTUC = upper tract urothelial carcinoma.

**Fig. 3:**
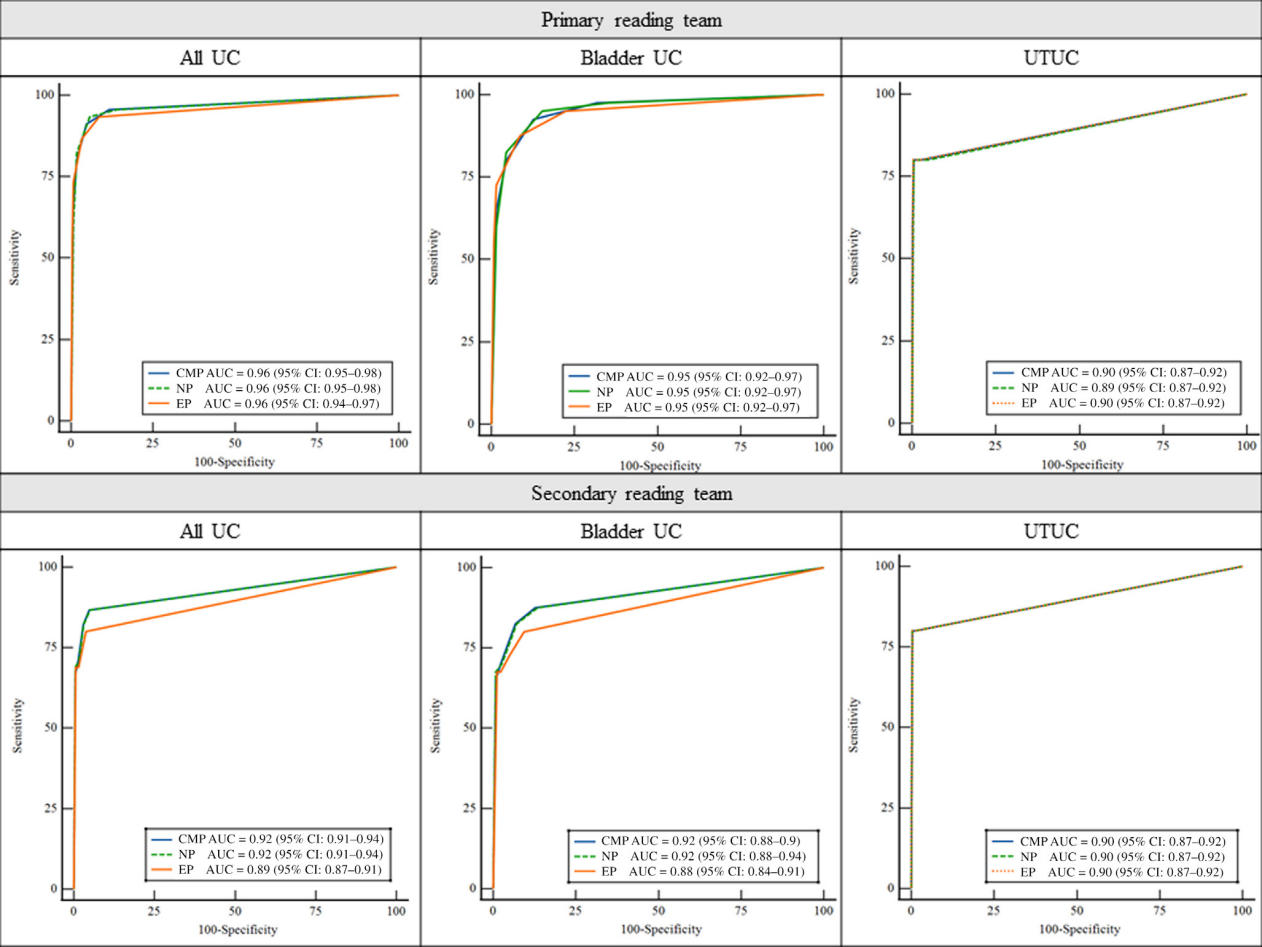
Receiver operating characteristics curves for the diagnostic performance of the different contrast phases of the control CT. AUC = area under the curve; CI = confidence interval; CMP = corticomedullary phase; CT = computed tomography; EP = excretory phase; NP = nephrographic phase; UC = urothelial carcinoma; UTUC = upper tract urothelial carcinoma.

The results of CT against the reference standard are shown in Table 2. For any UC, the control and experimental CT detection rates were 13.6% (95% CI: 10–18) and 13.3% (95% CI: 10–18), and the sensitivities were 93.3% (95% CI: 82–98) and 91.1 (95% CI: 79–97), respectively. The overall accuracy of the control versus experimental CT was 85% (95% CI: 80.6–88.6) versus 83.1% (95% CI: 78.5– 86.9). Thus, the difference was 1.9% (Table 3). The CI of the difference was calculated using the discordant pair proportions according to Liu et al. [12], resulting in 95% CI values of –2.8 to 6.7. Since the upper limit of the CI did not exceed 7.5%, noninferiority was demonstrated. There was no difference in AUCs between the control and experimental CT or between the different contrast phases of the control CT (Fig. 3). The number of patients needed to scan with the control CT to detect one additional UC compared with the experimental CT was 311.

**Table 2:** Results of the control and experimental CT against the reference standard

	Primary reading team				Secondary reading team			
			Control CT				Control CT	
			Neg	Pos			Neg	Pos
*Bladder and upper urinary tract*								
UC absent								
	Experimental CT	Neg	192	23	Experimental CT	Neg	229	15
		Pos	28	20		Pos	11	8
UC present								
	Experimental CT	Neg	2	2	Experimental CT	Neg	4	4
		Pos	1	40		Pos	4	33
*Bladder*								
UC absent								
	Experimental CT	Neg	202	22	Experimental CT	Neg	240	12
		Pos	25	19		Pos	9	7
UC present								
	Experimental CT	Neg	1	2	Experimental CT	Neg	3	4
		Pos	1	36		Pos	4	29
*Upper urinary tract*								
UC absent								
	Experimental CT	Neg	290	4	Experimental CT	Neg	290	6
		Pos	7	2		Pos	6	1
UC present								
	Experimental CT	Neg	1	0	Experimental CT	Neg	1	0
		Pos	0	4		Pos	0	4

CT = computed tomography; Neg = negative; Pos = positive; UC = urothelial carcinoma.

**Table 3:** Diagnostic performance of the control and experimental CT for detecting UC

	Primary reading team			Secondary reading team		
	Control CT (95% CI)	Experimental CT (95% CI)	Difference (95% CI)^a^	Control CT (95% CI)	Experimental CT (95%CI)	Difference (95% CI)^a^
*Bladder and upper urinary tract*						
Sensitivity	93.3 (82.1–97.7)	91.1 (79.3–96.5)	2.2 (–11.8 to 16.3)	82.2 (68.7–90.7)	82.2 (68.7–90.7)	0.0 (–17.5 to 17.5)
Specificity	83.7 (78.7–87.6)	81.8 (76.6–86.0)	1.9 (–4.9 to 8.6)	91.3 (87.2–94.1)	92.8 (89.0–95.3)	–1.5 (–6.6 to 3.5)
NPV	98.7 (96.1–99.5)	98.2 (95.4–99.3)	0.5 (–2.6 to 3.7)	96.8 (93.8–98.4)	96.8 (93.9–98.4)	0.0 (–3.7 to 3.6)
PPV	49.4 (39.0–59.8)	46.1 (36.1–56.4)	3.3 (–12.1 to 18.5)	61.7 (49.0–72.9)	66.1 (53.0–77.1)	–4.4 (–22.3 to 14.0)
FNR	6.7 (2.3–17.9)	8.9 (3.5–20.7)	–2.2 (–16 to 11.8)	17.8 (9.3–31.3)	17.8 (9.3–31.3)	0.0 (–17.5 to 17.5)
FPR	16.4 (12.4–21.3)	18.3 (14.1–23.4)	–1.9 (–8.6 to 4.9)	8.8 (5.9–12.8)	7.2 (4.7–11.0)	1.5 (–3.5 to 6.6)
AUC	0.96 (0.95–0.97)	0.94 (0.93–0.96)	0.02 (–0.01 to 0.05)	0.92 (0.90–0.94)	0.91 (0.89–0.93)	0.01 (–0.05 to 0.07)
Accuracy	85.0 (80.6–88.6)	83.1 (78.5–86.9)	1.9 (–2.8 to 6.7)	89.9 (86.1–92.8)	91.2 (87.6–93.9)	–1.3 (–5.3 to 2.7)
*Bladder*						
Sensitivity	95.0 (83.5–98.6)	92.5 (80.1–97.4)	2.5 (–11.8 to 17.1)	82.5 (68.1–91.3)	82.5 (68.1–91.3)	0.0 (–18.5 to 18.5)
Specificity	84.7 (79.9–88.5)	83.6 (78.7–87.5)	1.1 (–5.4 to 7.6)	92.9 (89.2–95.4)	94.0 (90.5–96.3)	–1.1 (–5.7 to 3.5)
NPV	99.1 (96.9–99.8)	98.7 (96.2–99.5)	0.4 (–2.3 to 3.3)	97.3 (94.5–98.7)	97.3 (94.5–98.7)	0.0 (–3.4 to 3.4)
PPV	48.1 (37.4–58.9)	45.7 (35.3–56.5)	2.4 (–13.6 to 18.3)	63.5 (49.9–75.2)	67.4 (53.4–78.9)	–3.9 (–22.9 to 15.7)
FNR	5.0 (–1.4 to 16.5)	7.5 (2.6–19.9)	2.5 (–17.1 to 11.8)	17.5 (8.8–32.0)	17.5 (8.8–32.0)	0.0 (–18.5 to 18.5)
FPR	15.3 (11.5–20.1)	16.4 (12.5–21.3)	–1.1 (–7.6 to 5.4)	7.1 (4.6–10.8)	6.0 (3.7–9.5)	1.1 (–3.5 to 5.7)
AUC	0.95 (0.92–0.97)	0.93 (0.90–0.96)	0.02 (–0.02 to 0.06)	0.91 (0.88–0.94)	0.90 (0.86–0.93)	0.01 (–0.06 to 0.08)
Accuracy	86.0 (81.7–89.5)	84.7 (80.3–88.3)	1.3 (–3.3 to 5.9)	91.5 (87.9–94.2)	92.5 (89.0–95.0)	–1.0 (–4.7 to 2.7)
*Upper urinary tract*						
Sensitivity	80.0 (37.6–96.4)	80.0 (37.6–96.4)	0.0 (–53.6 to 53.6)	80.0 (37.6–96.4)	80.0 (37.6–96.4)	0.0 (–53.6 to 53.6)
Specificity	98.0 (95.8–99.1)	97.0 (94.5–98.4)	1.0 (–1.9 to 4.0)	97.7 (95.3–98.9)	97.7 (95.3–98.9)	0.0 (–2.9 to 2.9)
NPV	99.7 (98.1–99.9)	99.7 (98.1–99.9)	0.0 (–1.8 to 1.9)	99.7 (98.1–99.9)	99.7 (98.1–99.9)	0.0 (–1.8 to 1.8)
PPV	40.0 (16.8–68.7)	30.8 (12.7–57.6)	9.2 (–30.9 to 47.7)	36.4 (15.2–64.6)	36.4(15.2–64.6)	0.0 (–40.0 to 40.0)
FNR	20.0 (3.6–62.5)	20.0 (3.6–62.5)	0.0 (–53.6 to 53.6)	20.0 (3.6–62.5)	20.0 (3.6–62.5)	0.0 (–53.6 to 53.6)
FPR	2.0 (0.9–4.3)	3.0 (1.6–5.6)	–1.0 (–4.0 to 1.9)	2.3 (1.1–4.7)	2.3 (1.1–4.7)	0.0 (–2.9 to 2.9)
AUC	0.89 (0.87–0.92)	0.90 (0.87–0.92)	0.01 (–0.002 to 0.006)	0.90 (0.87–0.92)	0.90 (0.87–0.92)	0.0003 (–0.003 to 0.004)
Accuracy	97.7 (95.4–98.9)	96.7 (94.1–98.2)	1.0 (–1.6 to 3.7)	97.4 (95.0–98.7)	97.4 (95.0–98.7)	0.0 (–2.7 to 2.7)

AUC = area under the curve; CI = confidence interval; CT = computed tomography; FNR = false negative rate; FPR = false positive rate; NPV = negative predictive value; PPV = positive predictive value; UC = urothelial carcinoma.

a All 95% CIs of the differences are calculated according to Newcombe [14] except the 95% CI of the difference in accuracy, which is calculated according to Liu et al. [12].

### 3.1 Upper urinary tract

For UTUC, the control versus experimental CT sensitivity was 80.0% versus 80.0%, specificity 98.0% versus 97.0%, accuracy 97.7% versus 96.7%, and NPVs 99.7% versus 99.7% (Table 3).

**Fig. 4:**
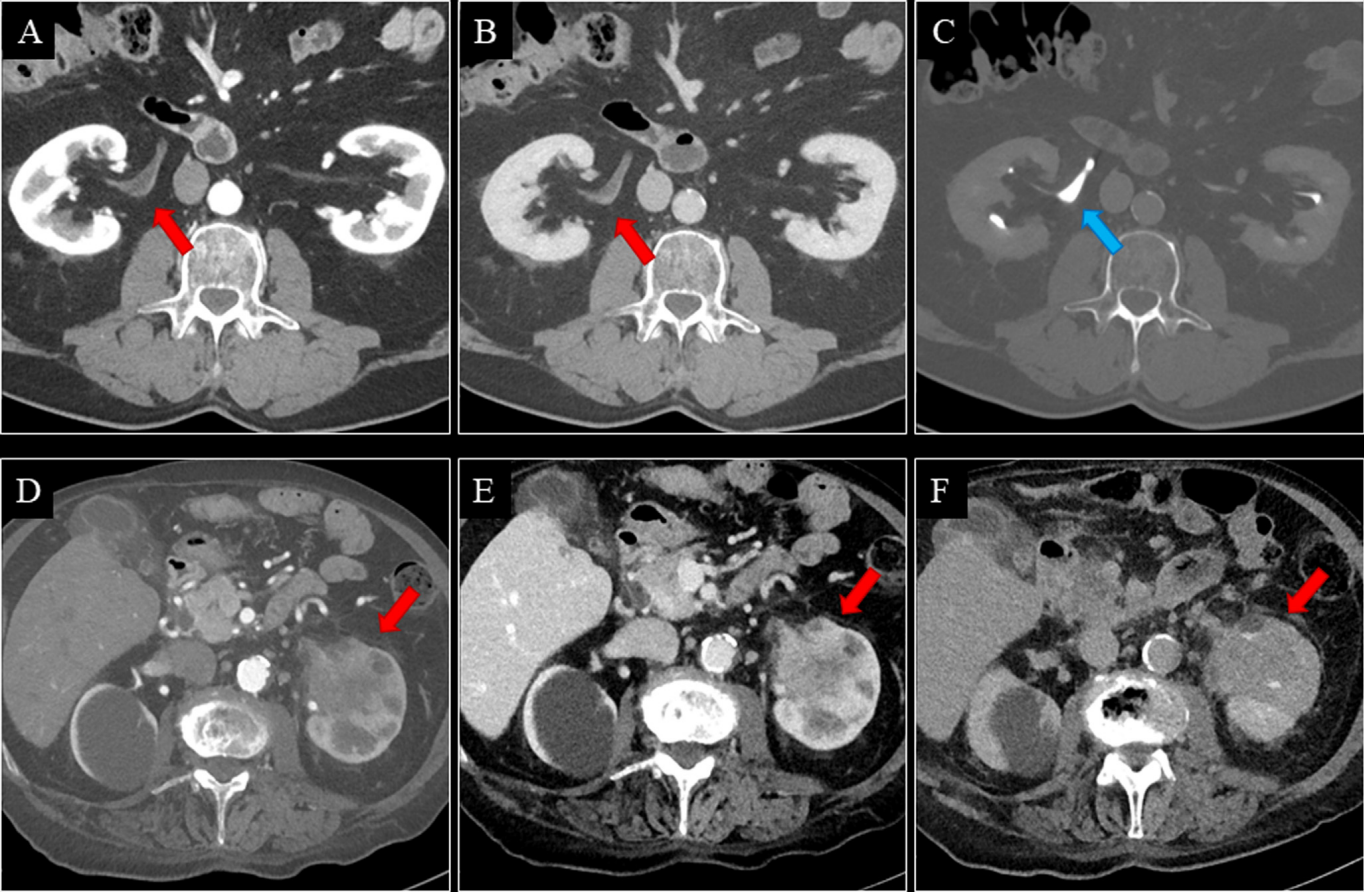
(A–C) A 68-yr-old male with a flat-growing pT1 tumour in the right renal pelvis. Both the control and the experimental CT were negative for renal pelvis UC in both reading teams. The tumour was detected at URS due to visible blood from the right ostium at cystoscopy. Retrospectively, a minimal contrast enhancement of the urothelium in the right renal pelvis (red arrow) was seen on axial CMP (A) and NP (B), but no filling defect was seen on axial EP (C; blue arrow). (D–F) An 87-yr-old male with a solid tumour in the left renal pelvis. Both the control and the experimental CT were positive for renal pelvis UC in both reading teams. The tumour (red arrow) was visible on all phases (axial CMP [D], NP [E], and EP [F]). The patient was unfit for URS, biopsy, and surgery, and received radiotherapy. CMP = corticomedullary phase; CT = computed tomography; EP = excretory phase; NP = nephrographic phase; pT = pathological tumour stage; UC = urothelial carcinoma; URS = ureterorenoscopy.

Of the five UTUCs, one flat-growing pT1 tumour in the right renal pelvis was missed on both the control and the experimental CT (Table 1). It was detected at URS due to visible blood from the right ureter at cystoscopy (Fig. 4A–C). One patient with positive CT (control and experimental) was unfit for URS, biopsy, and surgery, and received radiotherapy (Fig. 4D–F). In this case, the reference standard was defined as positive. No UTUCs were detected at URS performed solely due to Paris IV–VI.

### 3.2 Bladder

**Fig. 5:**
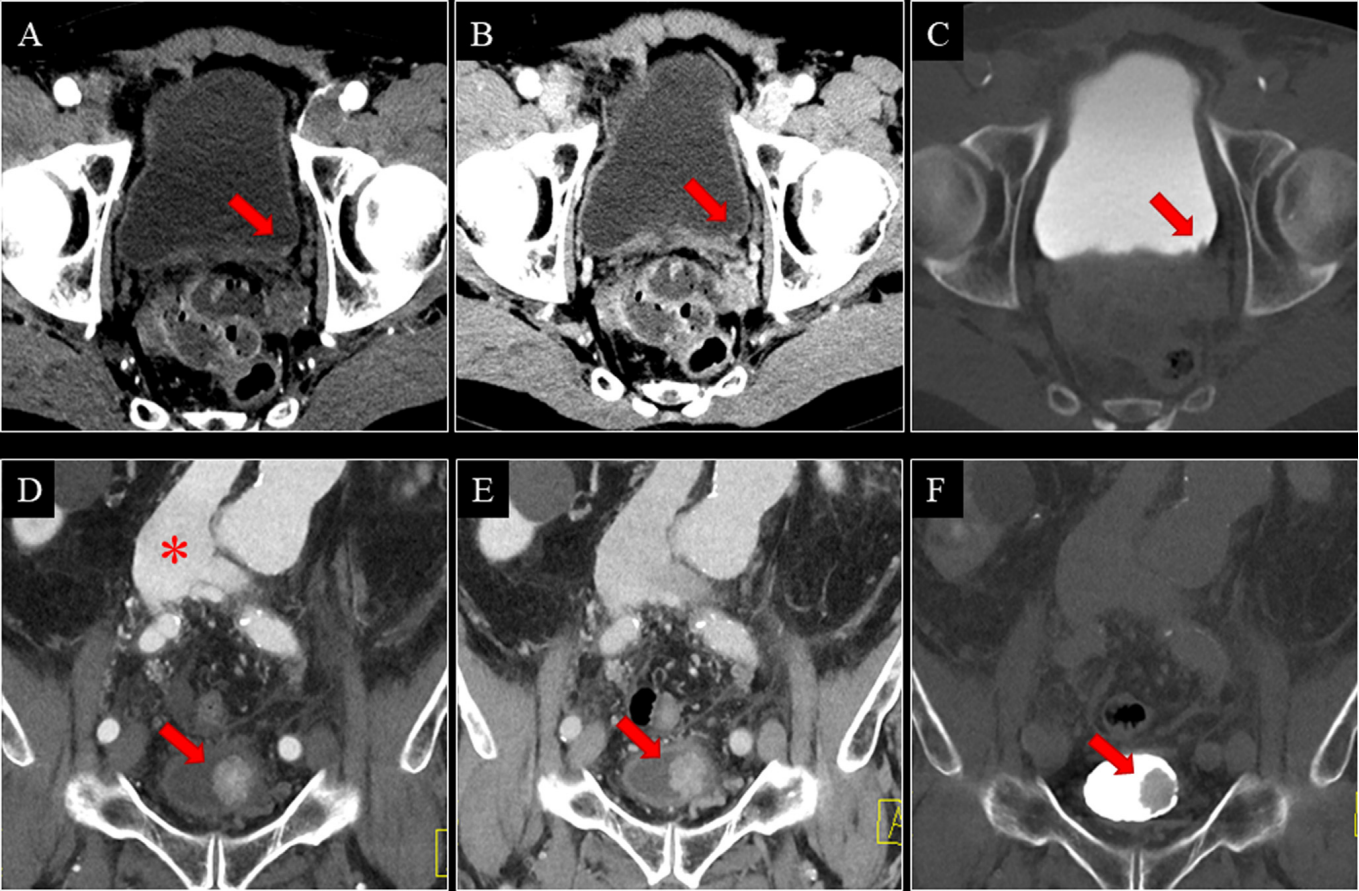
(A–C) A 73-yr-old male with a pTa tumour in the bladder. The experimental CT was negative for bladder UC in both reading teams. Retrospectively, the tumour (red arrow) was visible on all phases (axial CMP [A], NP [B], and EP [C]). The control CT was positive for bladder UC in both reading teams. (D–F) An 83-yr-old male with a solid tumour in the bladder. Both the control and the experimental CT were positive for bladder UC in both reading teams. The tumour (red arrow) was visible on all phases (coronal CMP [D], NP [E], and EP [F]). An ilioiliac fistula caused arterial filling of the inferior vena cava (red asterisk). Large vesical varices due to the ilioiliac fistula made the patient unfit for TURB, and he received radiotherapy. CMP = corticomedullary phase; CT = computed tomography; EP = excretory phase; NP = nephrographic phase; pT = pathological tumour stage; TURB = transurethral resection of the bladder; UC = urothelial carcinoma.

**Fig. 6:**
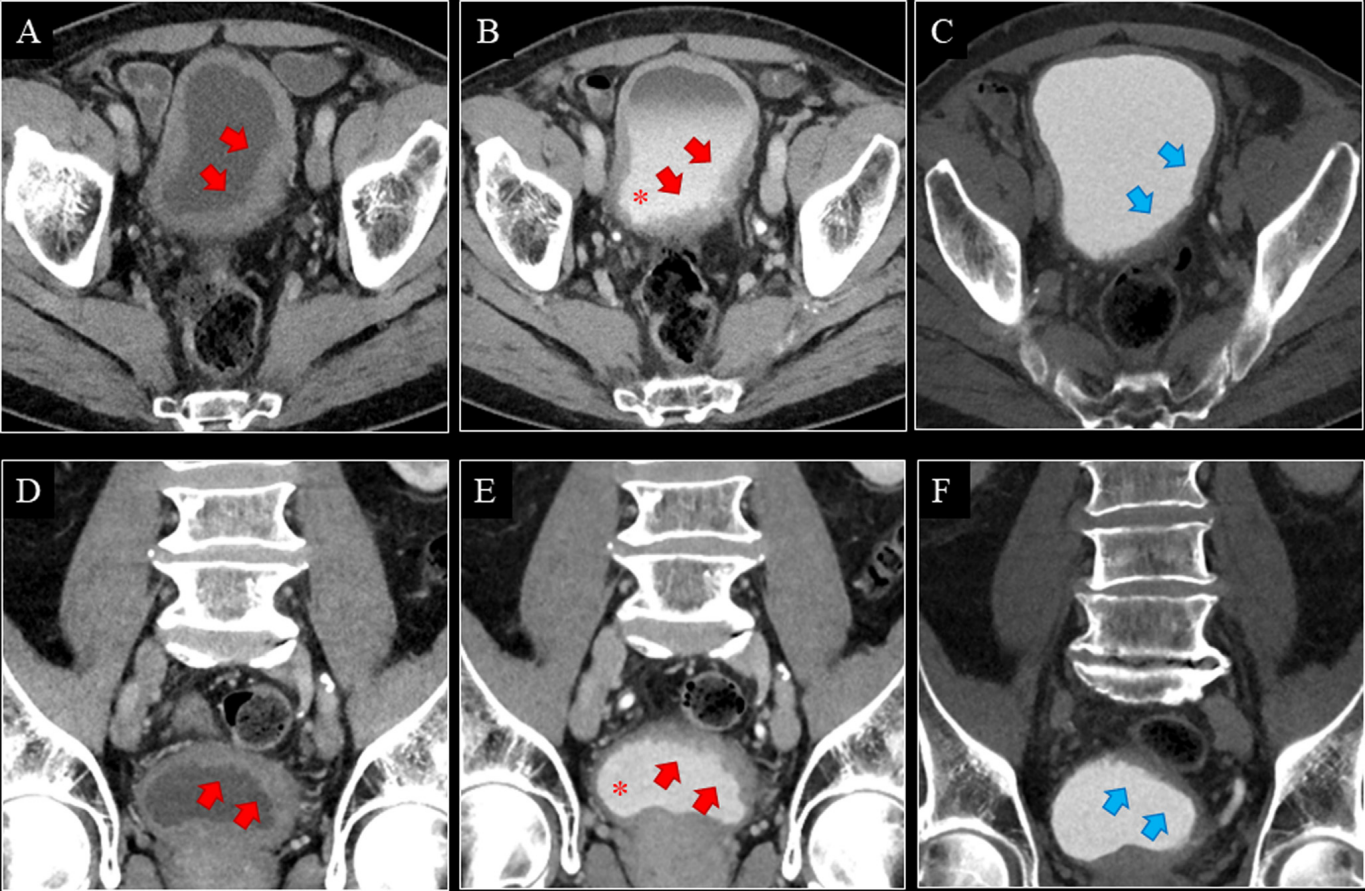
A 76-yr-old male with a flat growing pT1 tumour in the left bladder wall. For the primary reading team, the experimental CT was positive and the control CT was negative for bladder UC. For the secondary reading team, both the experimental and the control CT were negative for bladder UC. Retrospectively, the tumour (red arrow) was visible on (A and D) CMP, (B and E) NP, and (C and F) possibly EP (blue arrows).The contrast medium was accidentally injected subcutaneously. Consequently, the automatic bolus tracking was unsuccessful, and the CMP was started manually after 45 s. Then, the patient received a second contrast injection, which was successfully injected intravenously, and the NP was performed at 70 s after the threshold of the second contrast injection, 7 min after the first contrast injection. This is why secreted contrast was seen in the urine on the NP (red asterisk). A poorly filled bladder on CMP and NP, and a smoothed-out bladder wall on EP were interpreted as bladder hypertrophy only by some of the readers. CMP = corticomedullary phase; CT = computed tomography; EP = excretory phase; NP = nephrographic phase; pT = pathological tumour stage; UC = urothelial carcinoma.

For bladder UC, the control versus experimental CT sensitivity was 95.0% versus 92.5%, specificity 84.7% versus 83.6%, accuracy 86.0% versus 84.7%, and NPVs 99.1% versus 98.7%. Of the 40 bladder UCs, two pTa tumours were missed on the experimental CT only (Fig. 5A–C), one pT1 tumour was missed on the control CT only (Fig. 6), and one pTa tumour was missed on both the control and the experimental CT (Table 1). One patient with positive CT (control and experimental) was unfit for TURB and surgery, and received radiotherapy (Fig. 5D–F). In this case, the reference standard was defined as positive.

### 3.3 Secondary reading team

The differences in accuracy between the control and experimental CT were –1.3 (95% CI: –5.3 to 2.7) for detecting any UC, –1.0 (95% CI: –4.7 to 2.7) for detecting bladder UC, and 0.0 (95% CI: –2.7 to 2.7) for detecting UTUC (Table 3). There was no difference in AUCs between the control and experimental CT, or between the different contrast phases of the control CT (Fig. 3).

## 4 Discussion

To the best of our knowledge, this is the first prospective comparison of two different CT protocols in a population presenting with VH. The incidences of bladder UC and UTUC were 13.0% and 1.6%, respectively. This is in accordance with a recent review and shows that our study population is representative for patients presenting with VH [2]. The difference in overall accuracy for detecting UC in our study was 1.9% (95% CI: –2.8 to 6.7), demonstrating that the experimental (single NP) CT was noninferior to the control (four-phase) CT. Noninferiority was also demonstrated for the secondary readers, which indicates that single NP CT is also sufficient for general radiologists. Considering the minor difference in accuracy and the high “number needed to scan” (311), we believe that performing more than a single NP CT scan for detecting UC is excessive. For comparison, we identified only two prospective studies comparing the diagnostic performance of different CT protocols, that is, the NP versus EP [10], [16]. Both studies concluded that the NP had a higher detection rate than the EP and suggested that it is possible to use single NP CT for evaluating the bladder and upper urinary tract. However, since both studies included high-risk patients only, they are not necessarily representative of patients with VH.

The European Association of Urology does not provide any CT recommendations in the case of VH, and its guidelines are mainly relevant for patients with verified UC [17], [18]. Still, most centres routinely apply multiphase CT for investigating VH [19], [20]. The latest systematic review of the diagnostic performance of CT in the upper tract highlights the retrospective nature of the included reports as the main limitation of the study [21].

### 4.1 Upper tract

The accuracy for detecting UTUC was 97–98%, and there was no benefit of adding extra contrast phases to the NP. The NPVs were >99% for all readers, which is similar to previous studies [9], [10], [22]. This shows an excellent ability to rule out UTUC regardless of the CT protocol.

The sensitivity for detecting UTUC was only 80% regardless of the CT protocol since all readers missed one out of five UTUCs (Fig. 4A–C). The low prevalence of UTUC in patients with VH makes a prospective sensitivity analysis challenging since the low number of cases results in wide 95% CIs. Studies reporting sensitivity >80% are case-control, retrospective, or high-risk cohort studies, and therefore, are not comparable with those reporting on patients presenting with VH [7], [9], [10], [22], [23], [24].

### 4.2 Bladder

For the primary readers, the sensitivity of the control versus experimental CT for detecting bladder UC was 95.0% versus 92.5%, and the accuracy was 86.0% versus 84.7%. This is comparable with other studies that report sensitivities of 89–95% and accuracies of 80–97% [3], [4], [5], [25]. The limited accuracies seen in our study were mainly due to high false positive rates, indicating that positive CT cannot replace a diagnostic cystoscopy. The secondary reader pair had fewer false positives and higher accuracy at the expense of lower sensitivity. The NPVs were >97% for all readers, which is in accordance with previous studies, raising the question of whether negative CT has the potential to replace a diagnostic cystoscopy [3], [4].

### 4.3 Evaluation of the phases

We found no benefit of adding the CMP and EP to the NP. This supports previous studies favouring one-phase CT in the workup of VH [10], [16], [22], [23], [24]. To the best of our knowledge, no study has shown that the EP significantly improves the diagnostic accuracy of CT in patients presenting with VH. Still, the French Society of Genitourinary Imaging Consensus group published guidelines for CT urography (CTU) in 2019, recommending the EP as a split-bolus technique or as an isolated phase in conjunction with other phases [11]. In our opinion, the routine use of the EP represents a remnant from the era of intravenous pyelograms (IVPs), and the practice should be reconsidered.

### 4.4 Limitations

A noninferiority limit should not exceed the effect size of the active control, and it should be acceptable clinically [26]. We considered the effect size of CTU to be approximately 15% since previous studies have shown that CTU is 15% more accurate than IVP [27], [28]. Thus, our 7.5% noninferiority limit was well below the effect size of CTU. Moreover, we believe that the noninferiority limit is clinically acceptable since one-phase CT reduces radiation and resource consumption. A lower noninferiority limit would require a larger sample size, making the study harder to complete. After the study’s completion, the observed results are of main interest. Whether clinicians are willing to accept possibly 6.7% lower accuracy is a matter of discussion.

Since the primary role of CT is to rule out UTUCs, and most UCs in our study were bladder UCs, one could question the validity of the study in the upper tract. The low prevalence of UTUC in patients with VH is universal for all prospective studies and impossible to overcome. However, the difference in accuracy was smaller for the upper tract than for the bladder, and the 95% CIs were narrower. In our opinion, this shows that the study is sufficiently powered to also make conclusions in the upper tract.

All readers in our study were experienced radiologists, which raises the question of generalisability to less experienced readers. The secondary readers were less dedicated to uroradiology but achieved higher accuracies and smaller differences between the control and experimental CT than the primary readers. This supports that the NP is also sufficient for general radiologists, although the performance of less experienced readers is unknown.

## 5 Conclusions

The accuracy of single NP CT is not inferior to four-phase CT for detecting UC in patients with VH. In the routine workup of UC, single NP CT will reduce patient radiation and improve radiological capacity due to shorter examination and reading time.

  ***Author contributions:*** Kristina F. Galtung had full access to all the data in the study and takes responsibility for the integrity of the data and the accuracy of the data analysis.

  *Study concept and design*: Galtung, Rud, Sandbæk, Baco.

*Acquisition of data*: Galtung, Rud, Sandbæk, Baco, Lauritzen, Bay, Naas.

*Analysis and interpretation of data*: Galtung, Rud, Lauritzen, Sandbæk, Bay, Ponzi.

*Drafting of the manuscript*: Galtung, Rud, Lauritzen.

*Critical revision of the manuscript for important intellectual content*: Galtung, Rud, Lauritzen, Sandbæk, Bay, Ponzi, Baco, Cowan, Naas.

*Statistical analysis*: Galtung, Rud, Lauritzen, Ponzi.

*Obtaining funding*: None.

*Administrative, technical, or material support*: None.

*Supervision*: Rud, Sandbæk, Baco.

*Other*: None.

  ***Financial disclosures:*** Kristina F. Galtung certifies that all conflicts of interest, including specific financial interests and relationships and affiliations relevant to the subject matter or materials discussed in the manuscript (eg, employment/affiliation, grants or funding, consultancies, honoraria, stock ownership or options, expert testimony, royalties, or patents filed, received, or pending), are the following: None.

  ***Funding/Support and role of the sponsor:*** None.

  ***Acknowledgements:*** We would like to thank our CT radiographer team, led by Ms. Gøril Meland, for making this study possible. We also appreciate the essential contribution from Mr. Bjørn Næss and Mr Morten Hørthe (DIPS electronic patient record) who, without payment, designed and customised a study-specific database within our electronic patient record.
